# Conspecific recognition and aggression reduction to familiars in newly weaned, socially plastic mammals

**DOI:** 10.1007/s00265-015-1952-7

**Published:** 2015-06-20

**Authors:** Kelly J. Robinson, Sean D. Twiss, Neil Hazon, Simon Moss, Mike Lonergan, Patrick P. Pomeroy

**Affiliations:** Sea Mammal Research Unit, Scottish Oceans Institute, University of St Andrews, St Andrews, Fife, KY16 8LB UK; School of Biological and Biomedical Sciences, Durham University, South Road, Durham, DH1 3LE UK; Scottish Oceans Institute, University of St Andrews, St Andrews, Fife, KY16 8LB UK; Divison of Cardiovascular & Diabetes Medicine, University of Dundee, Ninewells Hospital & Medical School, Mailbox 2, Dundee, DD1 9SY UK

**Keywords:** Aggression, Grey seal, Mammal, Oxytocin, Pinniped, Recognition

## Abstract

Recognising conspecifics and behaving appropriately towards them is a crucial ability for many species. Grey seals (*Halichoerus grypus*) show varying capabilities in this regard: mother-pup recognition has been demonstrated in some geographical populations but is absent in others, yet there is evidence that individuals aggregate with prior associates. The recognition capabilities of newly weaned grey seal pups were investigated using class recognition trials within the habituation/dishabituation paradigm. Trials took place in pens, using pairs of individuals that either had previously cohabited (familiar) or that had never met before (stranger). Frequencies of olfactory and visual investigative behaviours (‘checks’) and aggressive interactions were recorded during trials. Familiar individuals recognised each other: paired strangers showed significantly more checks and aggressive interactions than were seen in trials pairing familiars. Oxytocin concentrations in post-trial plasma samples were analysed to investigate the underlying physiology modulating recognition abilities; however, no significant differences were detected between familiar or stranger trials. This study demonstrates that at a young age, grey seals can recognise individuals they have previously encountered. Recognition abilities in this species have adaptive value by allowing the reduction of costly aggressive interactions between familiar conspecifics, which is often cited as the first step towards the evolution of sociality in a species. This study is the first with wild subjects to find conspecific recognition abilities in a pinniped species outside of reproductive contexts. It demonstrates that even largely solitary species can be capable of recognition and pro-social behaviours that benefit them during times when they must aggregate.

## Introduction

Recognition between conspecifics is a vital first step to link past experiences to present companions during repeated interactions. Recognition allows behaviours ranging from aggression to affiliation to be directed towards particular individuals within a group or across periods of separation and is present in a wide range of animal taxa (Tibbetts and Dale 2007). Recognising a particular animal, or their status, allows an individual to exhibit appropriate behaviour in response to a conspecific, enabling the formation and maintenance of dominance hierarchies (Barnard and Burk 1979), mother-infant bonds (Cheney and Seyfarth 1980) and social groups (Sayigh et al. 1999). Recognition can occur at many levels, including identifying features such as an individual’s species or sex (Gwilliam et al. 2008), ‘class’ recognition to identify different dominance, familiarity, social group or relatedness classes and ‘true’ individual recognition (Tibbetts and Dale 2007). Studies documenting the different recognition abilities of species that live in social groups (e.g., Sharpe et al. 2013) or solitary species which maintain dominance hierarchies between competing individuals (e.g., Gherardi and Tiedemann 2004) are well represented in the literature. However, few studies have investigated recognition abilities in species that are largely solitary and form groups only during specific activities or times of year.

Pinnipeds spend much of their lives foraging at sea without a stable social environment. Despite this, most species must aggregate ashore in large numbers for short periods of time (Boness and Bowen 1996). Pinnipeds in reproductive contexts have provided examples of different types of recognition (reviewed in Insley et al. 2003). Class recognition between rival breeding males (Tripovich et al. 2008; Casey et al. 2013) and individual recognition between mothers and pups (Roux and Jouventin 1987; Charrier et al. 2002, 2003, 2009; Pitcher et al. 2010, 2011) has been identified. Moreover, in captive settings, individual recognition has been demonstrated in Pacific walrus (*Odobenus rosmarus divergens*, Charrier et al. 2011). There are also pinniped species that lack recognition abilities in similar contexts, such as the failure of Hawaiian monk seal (*Monachus schauinslandi*) mothers to recognise their own pups (Job et al. 1995). However, recognition between free-ranging individuals in non-reproductive contexts has never been demonstrated in any pinniped species, despite evidence of inter-annual associations between breeding females creating fine-scale social structure in breeding colonies (Pomeroy et al. 2005; Wolf and Trillmich 2007) and suggestions that offshore associations may occur while foraging (Lidgard et al. 2012). Grey seals (*Halichoerus grypus*) are phocids distributed over the north Atlantic, Baltic Sea and northern Russian waters. Although there is evidence that at least some adults in the UK recognise and actively associate with previously encountered conspecifics on breeding colonies (Pomeroy et al. 2005), mothers from the eastern Atlantic have been shown to fail to recognise their own pups (McCulloch et al. 1999; McCulloch and Boness 2000).

In this study, we use an experimental approach adapted from existing rodent recognition trials (Ferguson et al. 2001; Bielsky and Young 2004) to investigate the recognition abilities of newly weaned grey seals and to test the hypothesis that familiar animals exhibit fewer aggressive behaviours towards each other. By investigating both recognition and aggression between novel and familiar individuals, we can examine one aspect of the adaptive value of recognition abilities. Risky or energetically costly behaviours, such as aggression, can be reduced if individuals can recognise familiar conspecifics, thereby receiving a fitness benefit over individuals unable to alter their behaviour to different familiarity classes (Höjesjō et al. 1998). Reduction of aggression towards familiar individuals is often cited as a crucial step towards sociality (Kleiman and Eisenberg 1973; Wolf and Trillmich 2007). Therefore, documenting such behavioural shifts in species that are principally solitary, or contexts where reduced aggression to familiar individuals occurs in the wild reveals situations where simple sociality can evolve.

Along with documenting any differences in frequencies of aggressive behaviour between familiar or novel individuals, this study aimed to investigate physiological proximate causes of such changes. Oxytocin is a neuropeptide hormone that has been identified as a potential biomarker of social relationships (Crockford et al. 2014). Elevated levels of peripheral oxytocin have been observed after interactions between familiar dogs (*Canis lupus familiaris*, Nagasawa et al. 2009; Romero et al. 2014) and humans (*Homo sapiens*, Levine et al. 2007; Feldman et al. 2007), and there is substantial evidence that oxytocin acts as an important regulator of social aggression in mammals (Lee et al. 2009; reviewed in Anacker and Beery 2013), especially out-group aggression towards strangers (De Dreu 2012). We investigated whether changes in investigative or aggressive behaviour when subjects were with a familiar or novel individual was accompanied by peripheral oxytocin release.

## Materials and methods

### Study site

This study took place on the Isle of May grey seal breeding colony in Scotland (56° 11′ N, 02° 33′ W) from 12th November to 6th December, 2010. Additional plasma samples for comparison purposes were collected on the North Rona grey seal breeding colony in Scotland (59° 06′ N, 05° 50′ W) between 2nd October–1st November, 2009, and 3rd October–31st October, 2010.

### Study animals

Eight newly weaned grey seal pups were captured on the Isle of May in 2010 for a pilot study of the recognition trials, and once this was completed, a group of 12 newly weaned grey seal pups were captured for the main series of trials. All the study animals had weaned within 4 days of each other. Captured pups were placed into one of two holding pens built on the breeding colony to generate two groups of individuals that had previously encountered those in their own pen, but not those in the other pen. Pens were built on the periphery of the breeding colony with a stone wall separating the pens and breeding adults. The sex ratio in the holding pens during the main trials was even (three males to three females per pen); however, in the pilot study, only one female was used with seven males. Pups were defined as being weaned on the second consecutive day of being seen without their mother after a normal rearing period, based on daily observations.

Adult seals were not suitable for this study due to the practical difficulties associated with their large size and aggressive behaviour. Weaned pups were deemed a suitable substitute for adults as the oxytocin receptor system in brain regions associated with social behaviours are mature at weaning in rodents (Wang and Young 1997). There is evidence that there are sex differences (de Vries 2008) and individual differences (Olazabal and Young 2006) in the oxytocin receptor systems that develop in juvenile rodents; therefore, we controlled for individual identity and sex in all analyses. Using adult females in these trials would have enabled a direct comparison with mothers on breeding colonies, but this was not practical, nor would it have been possible to control for prior familiarity. The use of newly weaned animals instead of adults allowed us to take advantage of the 1–4 week post-weaning fast in this species (Reilly 1991) and allows us to test whether newly weaned individuals are capable of forming affiliations at that age as previously hypothesised (Pomeroy et al. 2005). Weaned pups can be captured, handled, transported and penned at this age, all crucial requirements for this study.

Upon capture, all pups were sexed, weighed and had a basal blood sample drawn using the methodology outlined below. Pups were weighed ± 0.2 kg on a spring balance (Salter Industrial Measurements Ltd., West Bromwich, UK). In accordance with previous studies using captive weaned grey seal pups to prevent the post-weaning fast period from being extended unnaturally (Bennett et al. 2007), pups weighing less than 30 kg initially were excluded from the study. Animals falling below 30 kg or 75 % of their capture mass would be released early from the trials. Mass was recorded every 3 days throughout the period of captivity. None of the study animals lost sufficient mass to warrant early release. To assist in identification, all animals were marked with gloss paint on the mid-dorsal region, receiving either a single letter or number depending on the pen in which they were held. All pups were released into the wild after they participated in either the pilot or main study, and time in captivity ranged from 5 days (pilot study) to 14 days (main study).

### Plasma sampling and analysis

Plasma samples were collected from free-ranging weaned pups on the Isle of May colony in 2010 (*n* = 12) and from the North Rona colony in 2009 (*n* = 8) and 2010 (*n* = 13) to generate basal oxytocin data for comparison to the oxytocin concentrations of the pen trial group. A series of plasma samples were also taken to test that being in a captive environment did not affect basal plasma oxytocin concentrations of trial individuals. Plasma samples were taken from seals at initial capture (*n* = 20), midway through the captivity period (*n* = 20) and post-release for as many individuals as could be located while free-ranging on the colony (*n* = 19). Post-release samples (2 days after release) were collected by searching the colony for study animals and using temporary physical restraint of animals in situ wherever they were found.

To obtain a plasma sample, animals were captured and physically restrained without chemical immobilisation. Samples were drawn from the extradural vein into two 10 ml lithium heparin vacutainers (®Becton, Dickinson and Company) and stored on ice until they could be spun at 1200 RCF for 10 min to separate the plasma. Plasma was then frozen at −20 °C. This sample collection and storage methodology has been shown to induce negligible oxytocin degradation over at least a 2-year period (Robinson et al. 2014).

Oxytocin release can occur both centrally and peripherally in response to physical and psychological stressors (Neumann and Landgraf 2012) and has been linked to restraint stress in rodents (Grippo et al. 2009). However, it has been shown that in grey and harbour (*Phoca vitulina vitulina*) seals, there is no significant difference in plasma oxytocin concentrations from samples obtained with the use of chemical immobilisation or manual restraint (Robinson et al. 2014). Additionally, plasma oxytocin concentrations have no relationship with the number of minutes of handling to obtain a sample (within the range encountered in these trials) as long as extracted plasma is used, as in this study (Robinson et al. 2014). Nevertheless, we attempted to obtain all samples within 5 minutes of the initial disturbance to the animal because of the reported short half-life of oxytocin in plasma (between 3 and 5 minutes in humans depending on reproductive state, Rydén and Sjöholm 1969). The time taken to obtain a sample and the time of day it was taken were recorded to allow these variables to be considered in the analysis.

Plasma was analysed for oxytocin using an ELISA (Assay Designs Inc., Ann Arbor, MI, USA) with each sample undergoing solid-phase extraction using Sep-Pak C18 columns (Szeto et al. 2011; Cool and DeBrosse 2003) prior to analysis following the methodology validated for detecting phocid plasma oxytocin (Robinson et al. 2014). After the plate was read using a BioTek ELx800 reader, the standard curve and assay results were fitted using the calibFit package (Haaland et al. 2011) in R version 2.9.2 (R Development Core Team 2008). Recovery rates for the extraction and ELISA procedure were 107.2 % (*n* = 10), intra-assay coefficient of variance (COV) for this assay was 6.2 % and inter-assay COV over the six plates used in this study was 10.3 %.

### Stranger/familiar trial protocol

Pens were constructed with permission from Scottish National Heritage and in accordance with UK Home Office guidelines on suitable temporary holding facilities for grey seals and were taken down once the trials were complete. Holding pens measured approximately 15 × 10 m each and contained pools of freshwater. Experiments took place in a separate 5 × 5 m trial pen out of sight of the holding pens. Holding pens were located adjacent to the breeding colony, separated by a stone wall, in a part of the island the breeding seals did not use. The holding pens were separated by 10 m to ensure that no interactions could occur through the fencing outside of the recognition trials and foliage growing naturally on the Isle of May blocked line of sight between the pens.

Regular observations on the pups while they were still with their mothers at various sites scattered throughout the Isle of May breeding colony allowed us to be confident that individuals in one pen had not come into contact previously with the individuals in the other. Two rounds of pen trials were conducted in this study. First, a pilot study using eight newly weaned pups penned into two groups of four was conducted for 5 days prior to the main trial. Subsequently, a group of 12 newly weaned pups were penned into two groups of six for 14 days. All study animals were rested for 1 day post-capture to acclimate to the holding pens and the five other individuals with them. Pen trials then commenced on the second day after capture. No individual was used in more than one trial per day, and pups had a rest day between each trial day.

**Fig. 1:**
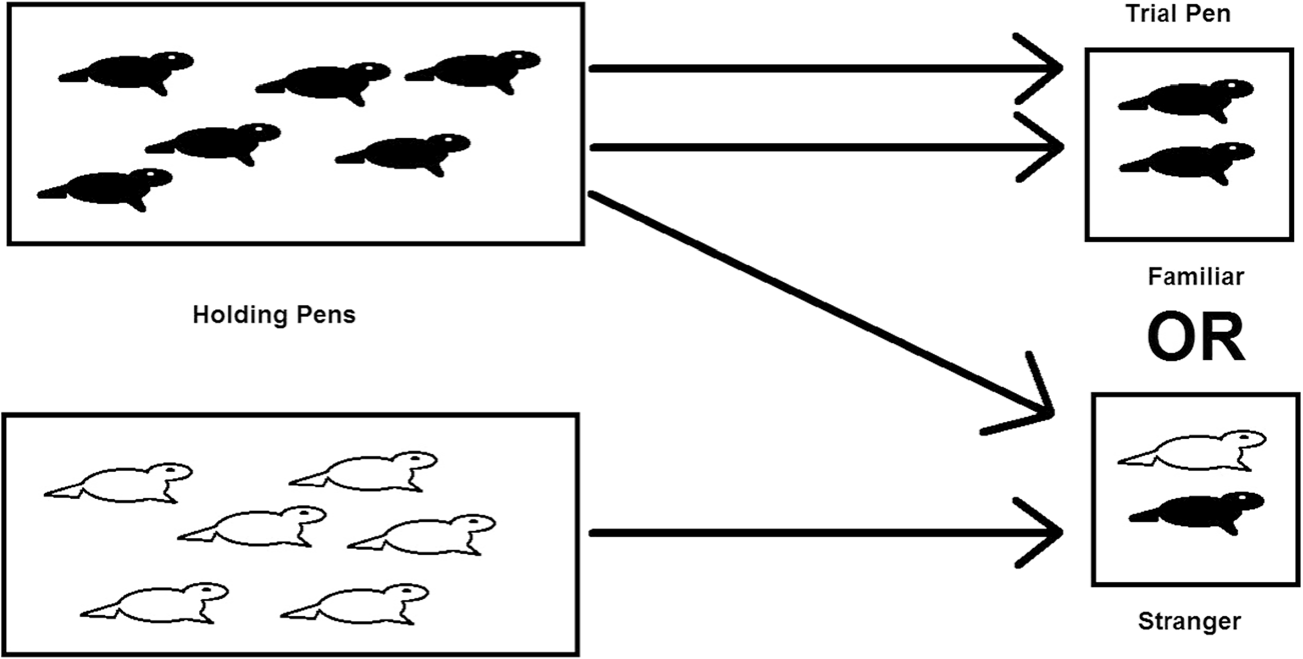
Paired pen trial design, with each holding pen containing six weaned seals and individuals from either the same pen (familiar trials) or from different pens (stranger trials) being used in the experiments

Each trial tested either a ‘stranger’ pair using one animal from each holding pen, or a ‘familiar’ pair using two animals from the same holding pen (Fig. 1). The two subjects required for a trial were captured in the holding pens and then carried while restrained in a bag to the trial pen at the same time. Mean time spent capturing and transferring pups from the holding pen to the trial pen was 2 minutes 24 seconds (SD = 1 minutes 6 seconds). The animals were introduced into the trial pen simultaneously in a standardised manner and trials were 1 hour long. Paired pen trials were recorded (video camera used: Panasonic HDC-TM60 HD 1920 × 1080) from a hide. After each trial individuals were returned to their original holding pen. In the pilot study, four trials were run per day with all study pups used once per day. In the main study, six trials were run per day with all study pups being used once per day. After a subset of both types of trials (familiar trials sampled: *n* = 16, stranger trials sampled: *n* = 10) plasma samples were taken immediately after the trial from both pups but before they were returned to the holding pens, to investigate post trial plasma oxytocin concentrations.

During the pilot study, eight trials took place over 5 days of captivity, consisting of four stranger and four familiar trials (*n* = 16 individual trial responses). During the main study, 40 trials took place over 14 days of captivity, consisting of 20 stranger trials and 20 familiar trials (*n* = 80 individual trial responses). This gave a total of 48 pen trials (*n* = 96 individual trial responses), with 24 familiar trials and 24 stranger trials (*n* = 48 individual trial responses per stranger or familiar type). Although the same individuals were used for multiple trials across their period in captivity, the same pairs of individuals were never recreated in any trials.

### Behavioural observations and decoding

Real-time video footage was decoded after all trials were completed to produce two types of metric for analysis: (i) frequencies of each behaviour type (affiliative, olfactory and visual investigative behaviours (‘checks’) or aggressive interactions between the trial animals) (Table 1) and (ii) the cumulative time in seconds spent within a threshold distance of one body length of each other. All distances were estimated visually in multiples of weaned pup length, which equates to approximately 1 m. One investigator (KJR) decoded all the videos for this study, and their error rate was assessed by decoding six different videos twice. The standard error for tallied frequencies of behaviours across the six videos ranged from 0 to 2 per video, and the standard error for cumulative time spent within 1 body length in trials ranged from 1 to 31 seconds.

**Table 1:** Behavioural categories and the specific behaviours used to classify weaned grey seal interactions in pen trials

Behaviour analysed	Specific behaviours included in category	Definition
Affiliation	Approach	Distinctive locomotion towards subjects, starting with the individuals separated by more than one body length and ending within one body length of each other.
Investigative (checks)	Visual check	Subject raises head above ground and makes a definite movement to look specifically at the other trial individual.
	Olfactory check	Subject approaches subject and extends the snout towards the other’s face, anogential region or any other part of the body with nares open and forward extension of the vibrissae. Physical contact with the other individual may or may not occur.
Aggressive	Open mouth threat	Head held low but above the ground, neck extended, mouth open. May be accompanied by other aggressive behaviours (see below).
	Vocalisation	Any hissing, growling or howls uttered while interacting with the other trial animal.
	Flippering	One or both fore flippers brandished rapidly at the other subject, may or may not come into physical contact.
	Lunge	Bite attempt, neck extends rapidly and then retracts without contact with the subject.
	Bite	Actual physical contact of one animal’s open mouth to any part of the other subject’s body, in a rapid and aggressive manner.
	Startle	Visible, physical startle response, typically a violent jumping or flinching action.
	Flee	Rapid locomotion away from the other subject, resulting in separation of more than one body length between the two individuals.

Colonially breeding adult grey seals typically maintain distances of approximately two adult body lengths (c. 4 m) between themselves and neighbours, and interactions between adults tend to only take place between individuals within that range (Redman 2002). We assume any individual within one body length of another would be sufficiently close to evoke a response from subjects. The most extreme reaction possible in the trial was biting, an infrequent behaviour. To ensure animal welfare was not compromised during the study, all trials were observed by a researcher who could intervene and separate subjects if necessary during a trial. This was not required during the study.

### Statistical analysis

All analysis was performed using the statistical package R 2.15.0 (R Development Core Team 2012). Generalised additive mixed models (GAMMs) (Wood 2006a) were used to investigate the following response variables: (i) the frequency of affiliation, checks and aggressive behaviours; (ii) the total cumulative number of seconds animals spent within one body length of each other. Biologically plausible predictor variables considered for inclusion in these models were the sex of the focal individual and time spent in captivity in days. The identities of both individuals in the trials were fitted as two random effect smooths (focal and response animal) (Wood 2006b) to control for pseudo-replication in the dataset due to use of the same individuals in multiple trials and to control for consistent individual differences in behaviour. The smoothing parameters were set by maximum likelihood to reduce the risk of over fitting associated with other methods (Wood 2011). All models of the frequencies of behaviours within the 1 hour trial were fitted with Poisson error distributions with log links using the multiple generalized cross-validation library mgcv (Wood 2012), whereas the model of time spent within one body length was fitted with a Gaussian distribution. All *p* values were calculated within the mgcv package, which allows for the complexities of the models. The models’ goodness of fit was examined by calculating *R*^2^ values, AIC scores, QQ and residual plots.

Another GAMM was used to analyse the response variable of post-trial plasma oxytocin concentrations. Biologically plausible predictor variables included in the selection process for this model were the sex of the focal individual, the time taken from ending the trial to plasma sampling and the time of day the sample was taken (recorded in ‘minutes since midnight’). The identities of both individuals in the trials were fitted as two random effect smooths (focal and response animal) to control for pseudo-replication in the dataset due to use of the same individuals in multiple trials and to control for possible individual or sex based variation in oxytocin receptor distributions in brain structures (de Vries 2008) that may impact on the oxytocin response generated by the trial. This model was fitted with a Gamma error distribution with log links using the multiple generalized cross-validation library mgcv. The full model and null model performed poorly in explaining the variance in the data, and therefore, model selection was conducted using backwards stepwise elimination of predictor variables through examination of *R*^2^ values, AIC scores, QQ and residual plots to identify the best model for the data.

Each of these analyses was treated as a separate investigation, without correction for multiple testing, because they considered separate response variables measured during the trials. However, it should be noted that even Bonferroni correction, which is appropriate for multiple analyses of a single set of response data, would only multiply the reported *p* values by five and not alter the conclusions of the study.

Basal plasma oxytocin concentrations were calculated for 12 free-roaming weaned pups from the Isle of May colony in 2010 and for 21 free-roaming weaned pups from North Rona in 2009 (8) and 2010 (13). These were compared using a one-way ANOVA to the study group’s plasma oxytocin concentrations at capture to check that the trial subjects were not unusual. The data were analysed after a natural log transformation as the original data were not normally distributed (Shapiro Wilk test, *p* = 0.01). Repeated measures ANOVA were performed on the longitudinal basal plasma oxytocin measurements taken from the study pups throughout the period of captivity to detect any changes caused by the pen and experimental environment.

## Results

### Behavioural frequencies during stranger/familiar trials

**Fig. 2:**
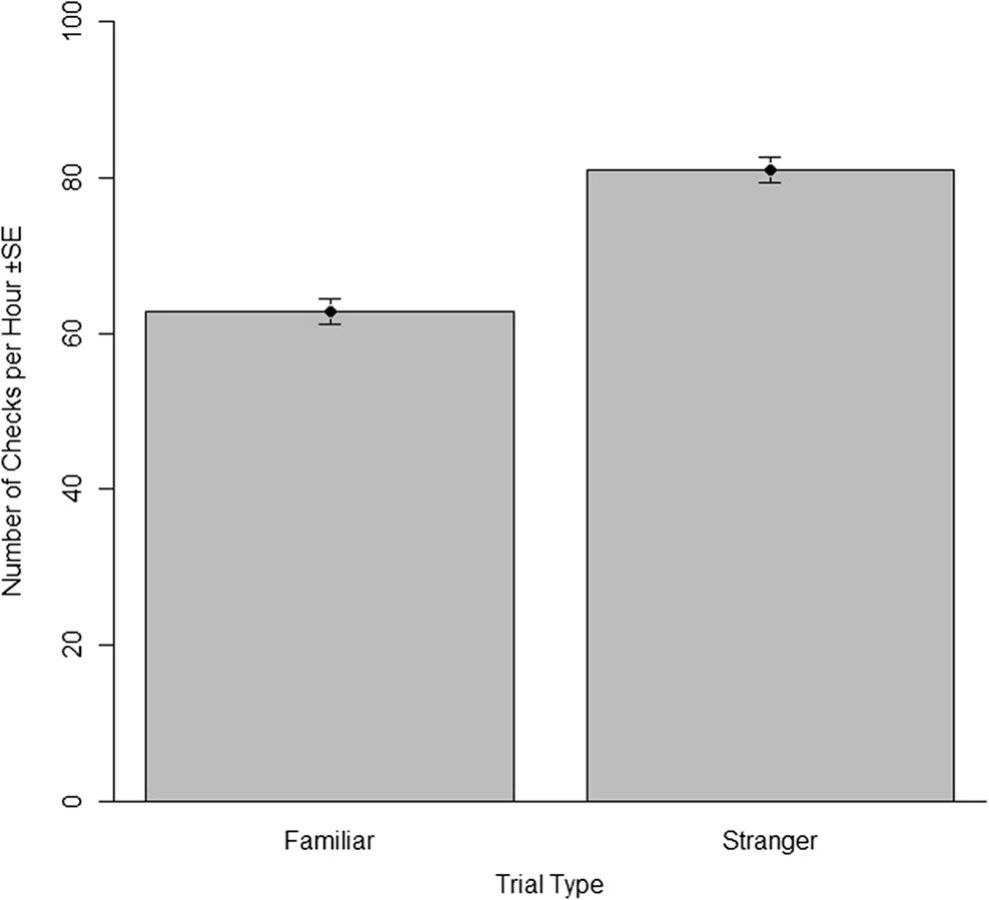
The frequency of checks across the two trial types, familiar (*n* = 48) and stranger (*n* = 48) with standard error bars

**Fig. 3:**
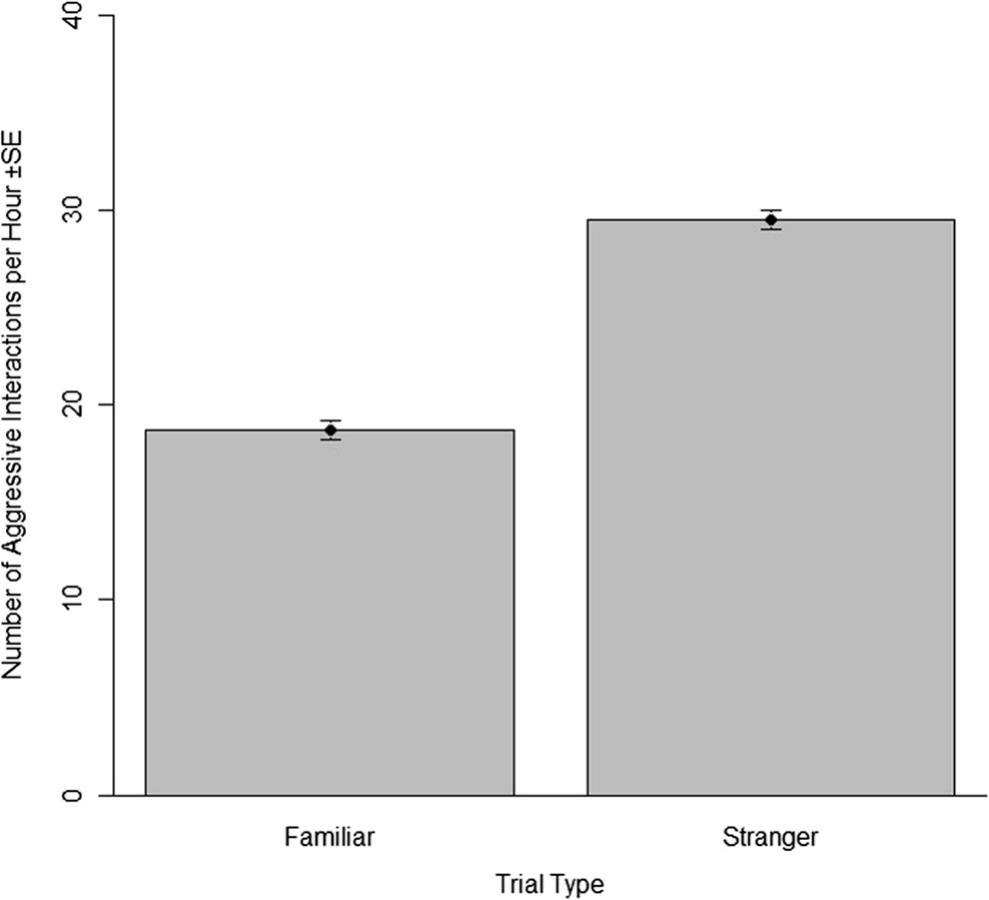
The frequency of aggressive behaviours across the two trial types, familiar (*n* = 48) and stranger (*n* = 48) with standard error bars

Pups performed significantly more checks and aggressive interactions during stranger trials (*n* = 48) compared to familiar trials (*n* = 48) (GAMM: *R*^2^ = 0.22, *p* < 0.001; GAMM: *R*^2^ = 0.73, *p* < 0.001, respectively) (Tables 2 and 3, Figs. 2 and 3). No predictor variables were eliminated from the model during the selection process. As time in captivity increased, pups made fewer checks and showed less aggression (*p* < 0.001 for each). Sex was not a significant variable in either model but was retained to report the full model. The individual identities of the trial animals were significant variables in both the check and aggression model (*p* < 0.001 for both, Table 4).

**Table 2:** Mean values (with standard errors) for the frequencies of approaches, checks, aggressive behaviour and the time spent within one body length of the response animal for each type of trial

Trial type	Approaches	Checks	Aggressive behaviours	Time within one body length (h:min:s)
Familiar (*n* = 48)	6.3 (±0.2)	62.7 (±0.1)	18.7 (±0.4)	00:37:20 (±00:02:55)
Stranger (*n* = 48)	8.04 (±0.08)	80.9 (±0.03)	29.5 (±0.05)	00:38:53 (±00:02:24)

**Table 3:** Model output from all GAMMs analysing behavioural responses in recognition trials with their standard errors, estimates and p values

Model: response variable	Predictor variables	Estimate	Standard error	*p* Value
Checks	Trial type (stranger)	0.2	0.03	<0.001
	Sex	−0.3	0.3	0.2
	Time in captivity	−0.04	0.003	<0.001
Aggressive interactions	Trial type (stranger)	0.4	0.05	<0.001
	Sex	−0.7	0.4	0.09
	Time spent in captivity	−0.06	0.006	<0.001
Approaches	Trial type (stranger)	0.2	0.08	0.08
	Sex	−0.4	0.4	0.3
	Time in captivity	−0.03	0.01	0.004
Time within one body length	Trial type (stranger)	59.6	144.4	0.7
	Sex	−334.4	263.6	0.2
	Time in captivity	−4.3	18.9	0.8

**Table 4:** Random effects model outputs from all GAMMs analysing behavioural responses in recognition trials with their standard deviations and *p* values

Model: response variable	GAMM random effect	Standard deviation	*p* Value
Checks	Focal Individual	0.5	<0.001
	Response Individual	0.4	<0.001
Aggressive interactions	Focal Individual	0.8	<0.001
	Response Individual	1.2	<0.001
Approaches	Focal Individual	0.8	<0.001
	Response Individual	0.2	0.03
Time within one body length	Focal Individual	432.7	0.06
	Response Individual	431.2	0.06

The number of affiliations and time spent within one body length of each other were similar in stranger and familiar trials (GAMM: *R*^2^ = 0.53, *p* = 0.08; GAMM: *t* = 0.4, *R*^2^ = 0.43, *p* = 0.7, respectively) (Tables 2 and 3). Sex was not a significant variable in either model but was retained to report the full model. Time in captivity (*p* < 0.005) and the identities of both the trial subjects (*p* < 0.05 for both) significantly affected the affiliation model, with fewer affiliations as time in captivity increased (Tables 3 and 4). There were no significant explanatory variables in the model for time spent within one body length (Tables 3 and 4).

### Plasma oxytocin concentrations after stranger/familiar trials

The plasma oxytocin concentrations detected in the weaned pups post-trial was not significantly different after stranger trials (13.2 SE ± 0.6 pg/ml) compared to those taken after familiar trials (11.8 ± 0.6 pg/ml) (GAMM: *t* = 1.9, *R*^2^ = 0.2, *p* = 0.06). Sex, time taken to sample and the time of day when sampled were not significant variables in the model and were eliminated during the selection process. Individual identities in the trials were not significant explanatory variables in the model (*p* > 0.05); however, they were retained as this increased the model’s ability fit to the data. The reduced model was reported as it accounted for the variance in the data better than the full and null models when assessed using *R*^2^ values, AIC values and from examination of QQ, residual and response/fitted values plots.

### Basal plasma oxytocin detection

**Fig. 4:**
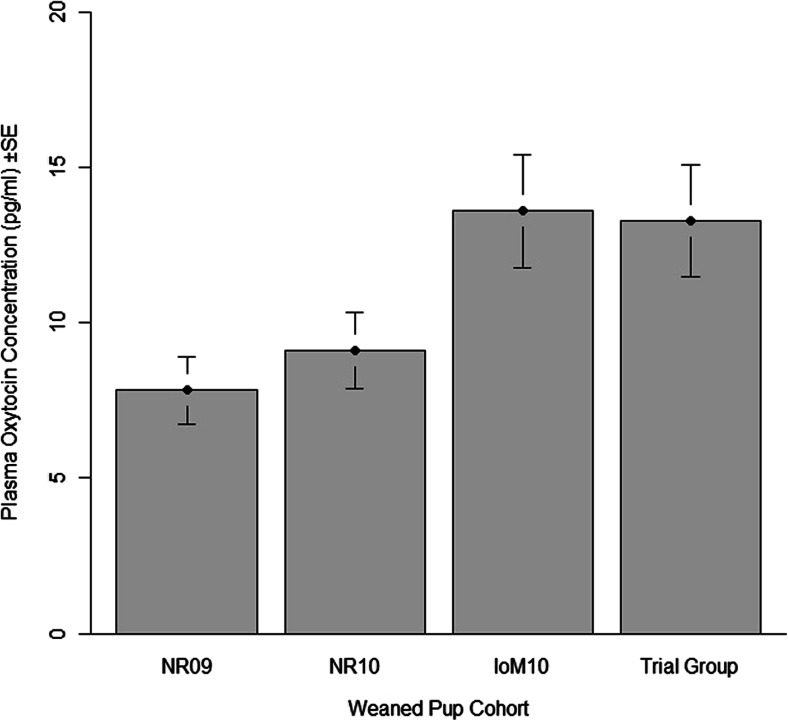
Mean plasma oxytocin (pg/ml) in the trial group compared to plasma oxytocin concentrations from free-roaming weaned pups from the North Rona colony in 2009 (NR09, *n* = 8) and 2010 (NR10, *n* = 13) and from the Isle of May in 2010 (IoM10, *n* = 12) with standard error bars

Basal plasma oxytocin concentrations were not the same in the groups of weaned pups from different breeding colonies (ANOVA: *F*_3,41_ = 7.09, *p* < 0.001). No statistically significant differences were detected between the 2010 trial group and the free roaming individuals from the Isle of May in 2010 (13.3 ± 1.8 and 13.6 ± 1.8 pg/ml, respectively, Tukey honest significant difference test, *p* = 0.99). There were also no significant differences observed between the 2009 and 2010 groups from the North Rona colony (7.83 ± 1.1 and 9.11 ± 1.2 pg/ml, respectively, Tukey honest significant difference test, *p* = 0.75). However, both North Rona groups differed significantly from both groups on the Isle of May (Tukey honest significant difference test, *p* < 0.05) (Fig. 4).

### Plasma oxytocin and captivity

Plasma oxytocin concentrations in individuals sampled before penning (14.2 ± 1.61 pg/ml) were not significantly different to those detected in samples taken at the midpoint of the period of captivity (11.8 ± 0.66 pg/ml) or those detected in samples taken 2 days after release from the pens (11.4 ± 0.74 pg/ml) (ANOVA: *F*_2,21_ = 2.319, *p* = 0.12).

## Discussion

Weaned grey seal pups in trials showed fewer checks and aggressive interactions when individuals were paired with a familiar compared to a stranger, indicating recognition between the two (Bielsky and Young 2004). The lower frequencies of both visual and olfactory checking shown by familiar grey seal weaned pups is also seen in rodents undergoing pen trials with familiar conspecifics (Ferguson et al. 2000), and indicates that the animals are aware they have previously investigated the other individual and do not need to conduct as extensive examinations of them. None of the pups with novel or familiar companions showed any significant change from basal plasma oxytocin concentrations. Therefore, while there is a significant detectable behavioural difference between responses to novel individuals and those previously encountered, we found no evidence in the plasma of grey seals that oxytocin release occurs during this change.

### Recognition and reduced aggression towards familiar individuals in the grey seal

This study confirms that grey seals are able to recognise other conspecifics as soon as they are weaned from their mothers. Additionally, they are able to adjust the aggressive behaviour they display to familiar individuals, preventing risky interactions that could result in injury to either party. There are other examples of reduced aggression occurring in largely solitary species for reasons other than familiarity, such as black bears (*Ursus americanus*) reducing aggression towards each other on human dump sites that provide a valuable food resource (Herrero 1983). Reduction of aggression towards individuals brought into repeated contact with each other due to a localised resource (e.g., feeding areas or aggregation of potential mates) has been described by the ‘Dear Enemy’ effect, where individuals with adjacent territories reduce aggression towards each other over time and repeated interactions (Fisher 1954). This type of recognition has been found in rival male pinnipeds guarding harems of females on breeding colonies (Tripovich et al. 2008; Casey et al. 2013). However, in our study, individuals were not protecting territories or resources, and familiar individuals performed fewer ‘checks’ on each other, which has been identified as a positive indicator of individual recognition abilities in rodents (Bielsky and Young 2004). The results of this study also cannot be explained by habituation to the trial environment or exposure to novel individuals. Throughout the study, individuals in stranger trials were consistently more investigative and aggressive than those in familiar trials. If habituation was responsible for these changes, the only significant variable influencing the models would be the time an individual spent in captivity, which was not the case. Therefore, we propose that the seals are capable of recognising familiar individuals with whom they have previously cohabited, rather than becoming habituated to the trial environment, protocols or exposure to novel individuals.

The ability to recognise and adjust behavioural frequencies towards conspecifics is potentially important for breeding grey seals. Grey seal mothers face a range of challenges within a breeding colony, and energetic output via aggressive behaviour towards neighbours is a necessity of living in a dense colony environment. A mother has limited resources to utilise while fasting on a colony, and the maternal resources expended in her pup during the intense 18-day rearing period (Pomeroy et al. 1999) is a major factor in the pup’s likelihood of surviving to weaning and its first year of life (Pomeroy et al. 1999; Hall et al. 2001). While fasting and nursing a pup, mothers can lose up to 40 % of their body mass at parturition and if an unusually large expenditure is made during one breeding season, the mother’s reproductive success will be negatively affected in the following year (Pomeroy et al. 1999). Therefore, the optimal strategy for expressing aggressive behaviour on a breeding colony would be to recognise real threats to the mother-pup pair and only direct aggression towards those. At the same time, an individual should recognise neighbours that are regularly encountered who do not harass or injure the mother-pup pair. Such recognition would prevent wasting limited resources on unnecessary aggression and reduce the risk of injury to the mother or her pup.

This study is the first to test directly for recognition between unrelated grey seals. Prior to this work, studies have only been able to predict the existence of recognition abilities in grey seals from looking at factors governing female pupping site choices (Pomeroy et al. 2000; Redman et al. 2001) or as a possible explanation of unexpected inter-annual patterns of affiliation (Pomeroy et al. 2005). The opportunity for repeated encounters between grey seal mothers exists because they are long-lived, exhibit site fidelity across breeding seasons (Pomeroy et al. 2000) and also show little variation in the date they give birth every year (Pomeroy et al. 1994). These characteristics of pupping behaviour mean that mothers return in successive seasons to pup adjacent to many of the same individuals, giving them repeated chances to interact with each other. While these aspects of behaviour of individuals within the colony allow for the existence of recognition, none provide direct evidence for its presence in grey seals as this study has done.

### Patterns of basal plasma oxytocin concentrations in grey seals

The concentrations of plasma oxytocin in study individuals were consistent throughout pre-capture, mid-point and post-capture sampling. Oxytocin is involved in the regulation of the hypothalamo-pituitary-adrenal (HPA) stress response (Tops et al. 2012) and has been linked to restraint stress in rodents (Grippo et al. 2009). Any stress caused by the pen environment and study protocol had the potential to alter the basal endocrine profiles of the study animals over time, introducing an undesired variable into our experiment. However, we found no evidence to indicate that this occurred during these trials. Furthermore, there was no difference between basal plasma oxytocin concentrations of the trial group and free-roaming weaned pups on the Isle of May, indicating that we had not sub-sampled an unusual and unrepresentative group for this study. However, there were differences between the basal concentrations from the North Rona and Isle of May colonies. While it is possible that differences in basal plasma oxytocin concentrations exist between the two colonies, the differences are more likely to be due to differences in the time individuals were weaned before sampling on North Rona compared to the Isle of May. As there was no way to tell how long the free-roaming weaned pups had been without their mothers, this could not be controlled for in this study. There is greater variability in plasma oxytocin concentrations in recently weaned pups compared to those without their mothers for 1 to 2 weeks and newly weaned pups have much higher plasma oxytocin concentrations than those weaned for over a week (Robinson 2014).

### Lack of change in plasma oxytocin concentrations across stranger and familiar encounters

This study found no post-trial peaks in plasma oxytocin across both stranger and familiar trials. To interpret this result, we must first consider whether our experimental design failed to generate conditions suitable for the occurrence of plasma oxytocin peaks, or if the protocol failed to detect peaks. Studies have shown rises in peripheral oxytocin as quickly as 15 min after positive social interactions, especially between individuals with an existing social bond (Odendaal and Meintjes 2003; Miller et al. 2009; Nagasawa et al. 2009; Crockford et al. 2013, 2014; Wittig et al. 2014). It is possible that while grey seals are capable of peripheral oxytocin release during interactions with associates, the trial environment used in this study did not result in any positive associations between individuals within the same holding pen. Although all the pups had cohabited with each other for the same length of time, there was no way to control for how much time they spent familiarising themselves with the other pen occupants, or whether the outcome would be a positive association between the two or a negative rejection. If no positive associations were formed between individuals, this may explain why no elevated plasma oxytocin concentrations were detected. However, as familiar individuals reduced aggression towards each other, it seems unlikely that negative associations were present throughout all of our familiar trials.

It is also possible that elevation of oxytocin concentrations in the periphery occurs in response to a specific interactive behaviour, such as food sharing in chimpanzees (Wittig et al. 2014), and that by sampling after a period of interactions our study may have missed capturing such peaks due to the rapid clearance rate of oxytocin from circulation (Rydén and Sjöholm 1969; Leake et al. 1980). There are no documented forms of pro-social behaviour between individual grey seals other than mothers caring for pups; therefore, in our study, it was not possible to sample after such behaviours. Significant rises in peripheral oxytocin concentrations have been recorded after a period of interaction between two familiar individuals (e.g., 30 min of interaction between dogs and their human owners, Nagasawa et al. 2009). Our sampling protocol was appropriate given this species limited social behaviour.

It is possible that plasma oxytocin peaks do not occur in interacting familiar grey seals. Phocid seals must be able to exhibit social plasticity as throughout their lives they occupy both extremes of the social range, from being solitary to living in large aggregations. Oxytocin acts as part of a conditioned positive feedback loop, released during positive social interactions which triggers the release of neurotransmitters involved in reward or pleasure pathways in the brain (e.g., in voles, *Microtus ochrogaster*, Gingrich et al. 2000). Oxytocin release, therefore, promotes the formation of stable pairs or groups that are then maintained for a period of time after the individual’s first encounter. This has obvious benefits for maintaining associations in highly social species that rely on group living for their survival, or between mothers and dependent offspring. However, there is no evidence currently to indicate that grey seals of any age must maintain associations while foraging at sea. Oxytocin release during interactions between familiar but non-filial individuals outside of the breeding season could be considered inappropriate, as it would encourage social behaviour or associations that are not necessary for an individual’s survival. Therefore, in grey seals, oxytocin’s role may be limited to the mother-pup bond, as plasma oxytocin concentrations have been correlated to the expression of some maternal behaviour in grey seals (Robinson 2014). Naturally elevated plasma oxytocin concentrations that occur when a mother is with her dependent pup may impact on a mother’s behaviour towards other conspecifics on a breeding colony (Robinson 2014). Nevertheless, our results emphasise that all grey seals posses the ability to modify their behavioural output towards familiar individuals without peripheral concentration changes of oxytocin.

## Conclusions

Reduction of aggression towards familiar individuals is often cited as a crucial step towards sociality (Kleiman and Eisenberg 1973; Barrett et al. 2002; Estevez et al. 2003). This study shows that grey seals are able to recognise individuals with whom they have previously cohabited and are capable of reducing the frequency of costly aggressive interactions with familiars. In mothers with dependent pups, these abilities should confer a selective advantage as they reduce costly aggressive behaviour, allowing increasing expenditure in pups. This provides a reason for seals retaining recognition abilities despite supposedly leading mostly solitary lives outside of colony environments. No changes in peripheral oxytocin were detected across the stranger and familiar trials. This could be due to limitations of the experimental methodology, or it could be because peripheral oxytocin release does not occur during conspecific recognition in grey seals. Oxytocin release may only occur to reinforce associations that are essential for the survival or reproductive success of an individual.
